# Establishing a valid cohort of patients with acromegaly by combining the National Patient Register with the Swedish Pituitary Register

**DOI:** 10.1007/s40618-023-02217-x

**Published:** 2023-10-18

**Authors:** J. Robèrt, E. Tsatsaris, K. Berinder, L. Bonelli, P. Burman, P. Dahlqvist, C. Höybye, D. S. Olsson, O. Ragnarsson, K. Vouzouneraki, A.-K. Åkerman, B. Ekman, B. Edén Engström

**Affiliations:** 1 Departments of Endocrinology in Linköping and Norrköping, Linköping University, Linköping, Sweden; 2 Department of Health, Medicine and Caring Sciences, Linköping University, Linköping, Sweden; 3 Department of Medical Sciences, Endocrinology and Mineral Metabolism, Uppsala University Hospital, Uppsala University, Uppsala, Sweden; 4 Department of Endocrinology, Karolinska University Hospital, Stockholm, Sweden; 5 Department of Molecular Medicine and Surgery, Karolinska Institutet, Stockholm, Sweden; 6 Department of Endocrinology, Skåne University Hospital Malmö, Lund University, Malmö, Sweden; 7 Department of Public Health and Clinical Medicine, Umeå University, Umeå, Sweden; 8 Department of Endocrinology, Sahlgrenska University Hospital, Göteborg, Sweden; 9 Department of Internal Medicine and Clinical Nutrition, Institute of Medicine at Sahlgrenska Academy, University of Gothenburg, Göteborg, Sweden; 10 Cardiovascular, Renal and Metabolism (CVRM), BioPharmaceuticals R&D, AstraZeneca, Gothenburg, Sweden; 11 Wallenberg Center for Molecular and Translational Medicine, University of Gothenburg, Göteborg, Sweden; 12 Department of Internal Medicine, Örebro University Hospital, Örebro, Sweden; 13 Faculty of Health and Medical Sciences, Örebro University, Örebro, Sweden

**Keywords:** Acromegaly, Patient register, ICD codes, Incidence

## Abstract

**Purpose:**

The aim of this study was to establish a valid national cohort of patients diagnosed with acromegaly by combining data from the general National Patient Register (NPR) and the disease-specific Swedish Pituitary Register (SPR).

**Methods:**

Patients ≥ 18 years of age at diagnosis of acromegaly reported from 1991 to 2018 who were registered in the NPR and/or SPR were included. The diagnosis of acromegaly was considered correct for patients identified in both registers or confirmed through chart review. Medical records were reviewed in two of Sweden´s six health care regions if the patient was reported only in the NPR. An algorithm for the NPR, with criteria requiring multiple diagnosis registrations and tumour and/or surgery codes, was constructed to reduce the number of patients to review in the remaining four regions.

**Results:**

A total of 1866 patients were identified. Among these, 938 were reported in both registers. After application of the algorithm and chart review, the diagnosis was confirmed for 83 of the 906 patients found only in the NPR. Among 22 patients only registered in the SPR, a review of medical records confirmed acromegaly in 13. This resulted in a total of 1034 cases with acromegaly during the study period. The incidence rate of acromegaly in Sweden 1991–2018 was calculated to 4.0/million/year in the entire population and 5.1/million/year among subjects ≥ 18 years of age.

**Conclusion:**

The combination of the SPR and NPR established a valid cohort of patients diagnosed with acromegaly and increased the estimated incidence in Sweden.

## Introduction

Acromegaly is a disease resulting from excess growth hormone (GH) derived from a pituitary tumour that causes growth of soft tissue and several associated conditions, such as sleep apnoea, arthropathy, hypertension, left ventricular dysfunction and diabetes [1].

Through a unique personal identity number (PIN), citizens of Sweden are registered in one or several mandatory or voluntary disease registers when seeking health care [2, 3]. Through this number, data can also be crosslinked between different registers and later be used for review of medical records [2, 4].

The National Patient Register (NPR) is a national public authority register and is managed by the National Board of Health and Welfare [5]. The specificity of diagnoses in the NPR is limited due to its automatic reporting. A review found a positive predictive value (PPV) of 85–95% and high (> 90%) sensitivity for most diagnoses in the inpatient part of the NPR [3], but validity for all parts of the register varies depending on the diagnosis sought. For instance, a recent Swedish study on Cushing’s disease found that 58% of the diagnoses were correct [6].

National quality registers are often diagnosis specific and are supported by an organization of health care professionals and patient representatives [7]. The Swedish Pituitary Register (SPR) is a quality register for diseases of the pituitary gland [8]. In a previous national study based on data from the SPR, the annual incidence rate of acromegaly in Sweden was 3.7 cases per million per year [9]. A similar incidence rate of 3.5 cases per million per year was found in a Swedish regional study [10]. However, the SPR is unlikely to include 100% of all Swedish acromegaly patients, resulting in a false low incidence.

Our aim was to create a valid cohort of all Swedish patients diagnosed with acromegaly from 1991 to 2018 by combining data from the NPR and the SPR. The secondary aims were to develop an algorithm that would result in a reduced number of patient charts that needed manual review to confirm the diagnosis and to calculate the national incidence of acromegaly.

## Subjects and methods

### Data sources

#### The national patient register

**Fig. 1:**
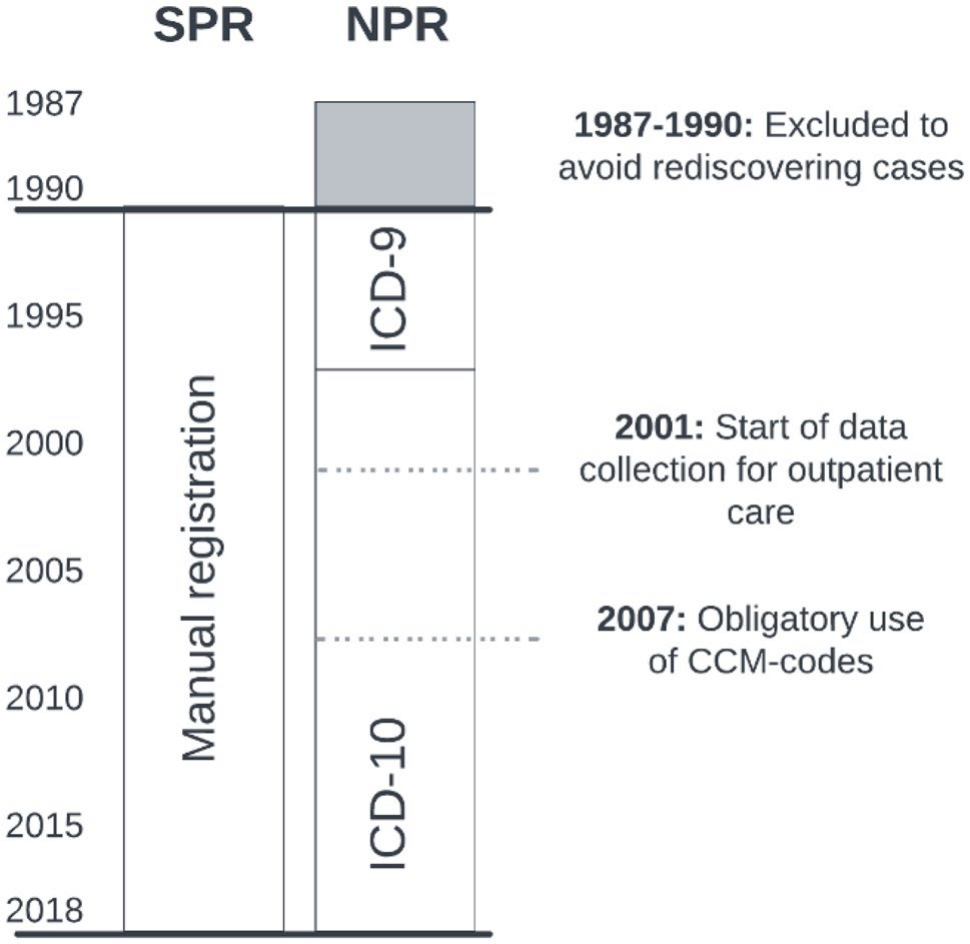
Timeline describing the NPR and SPR. Patients with diagnosis registration 1987–1990 were excluded to avoid rediscovering patients diagnosed previously

The NPR compiles all diagnoses set by health care staff in connection with all in- and outpatient visits to specialized health care. The register uses the Swedish version of the International Classification of Diseases (ICD) and was founded in 1964. Registrations from outpatient clinics started in 2001 (Fig. 1). Data from the Classification of Care Measures (CCM) regarding surgery and other medical interventions are also appended.

#### The Swedish pituitary register

The SPR was established in 1991 and is operated by the Regional Cancer Center (RCC) Stockholm-Gotland. The SPR is financially supported by the Swedish government, with the objective of evaluating given therapy for patients with pituitary tumours and ensuring equal care across Sweden’s six health care regions. All data are registered manually by treating physicians.

#### Study design

*Step 1* The search in the NPR included all patients who had received diagnosis codes for acromegaly in the ICD-9 or -10 (253A or E22.0, respectively) from 1 January 1987 until 31 December 2018. In addition, patients who had been registered as having acromegaly in the SPR from 1991 until 2018 were also identified. Patients who received their first diagnosis registration between 1987 and 1990 were excluded to avoid the selection of previously diagnosed patients. The inclusion criteria were a diagnosis of acromegaly in any of the registers, first diagnosis between 1991 and 2018 and ≥ 18 years of age at the time of diagnosis.

The patients were divided into three groups:

(NPR + SPR group) Patients registered as having acromegaly at least once in both the NPR and the SPR.

(SPR only group) Patients registered as having acromegaly in the SPR only.

(NPR only group) Patients registered as having acromegaly in the NPR only.

*Step 2* Patients who were present in both registers (NPR + SPR group) were defined as true cases, since both manual registration in the SPR as well as a recorded visit to a health care professional with the same diagnosis in the NPR was needed.

For patients registered only in the SPR (SPR only group), a review of medical records was performed to confirm or reject the diagnosis. A review of medical records was performed by a nonblinded physician and was based on an assessment of the complete clinical findings, including medical notes, symptoms, biochemical testing, radiology, and histopathology reports.

Patients registered only in the NPR (NPR only group) who had received their diagnosis of acromegaly in the south-eastern or mid Sweden health care regions (comprising 3.2 million of Sweden’s 10.3 million inhabitants in 2019) were validated through a review of medical records to confirm or reject the diagnosis.

Characteristics of NPR diagnosis code combinations of the patients with a diagnosis confirmed through review of medical records in these two regions were used to create an algorithm with the aim of retaining high sensitivity and excluding as many false-positives as possible. This method also had to detect patients coded in both the ICD-9 and ICD-10 systems, as well as both surgically and non-surgically treated patients. The algorithm was constructed based on tables of PPV and sensitivity of different variables in the NPR. The algorithm was validated by applying it to cases in the NPR + SPR group (i.e., defined as a diagnosis of acromegaly) and evaluating its sensitivity to correctly identify these patients.

*Step 3* The algorithm was then applied to the registrations for the patients in the NPR only group in the remaining four health care regions in Sweden. Cases selected by the algorithm underwent a review of medical records to confirm acromegaly diagnosis, as detailed above. Cases not selected by the algorithm and patients for whom medical records could not be found were excluded from the analysis.

Subjects in the western region only underwent review of medical records if they had a visit registered at Sahlgrenska University Hospital in Gothenburg. This patient population has been thoroughly researched previously, and charts for all patients with a diagnosis code for acromegaly in the NPR were reviewed in 2014 for another project [10].

This study and the data collection it entailed have been approved by the Regional Ethics Review Board, Uppsala, Sweden (No. 2017/475). Patients in the SPR must be informed and given the option to opt out of the register at any time. No informed consent is required for registration in national quality registers.

### Statistical analysis

Incidence data were calculated using Microsoft Excel (Microsoft Corporation, Redmond, WA.). All other data analyses were performed with R version 4.2.1 (R Foundation for Statistical Computing, Vienna, Austria) in RStudio (Posit Software, PBC, Boston, MA.). Incidence rates were calculated using population data from Statistics Sweden, and a Poisson distribution was assumed when calculating confidence intervals (CIs). The total number of person-years in the population in the nation and regions were obtained and used for incidence calculations. For the calculation of adult incidence, only individuals ≥ 18 years of age were included in the at-risk population. For calculation of total incidence, the entire population was included in the at-risk population. For comparison purposes, age standardised incidence rate given as rate /10^6^ inhabitants (95% CI), was calculated using the Swedish 2000 standard population and the WHO standard population as a reference, respectively. The local region of Halland is divided between southern and western health care regions, and population data were obtained directly for the municipalities corresponding to the respective health care region. For temporal changes in incidence, a linear model was fitted with yearly incidence as the dependent variable. For comparison of categorical variables, Fisher’s exact test was used.

## Results

### Study Population

**Fig. 2:**
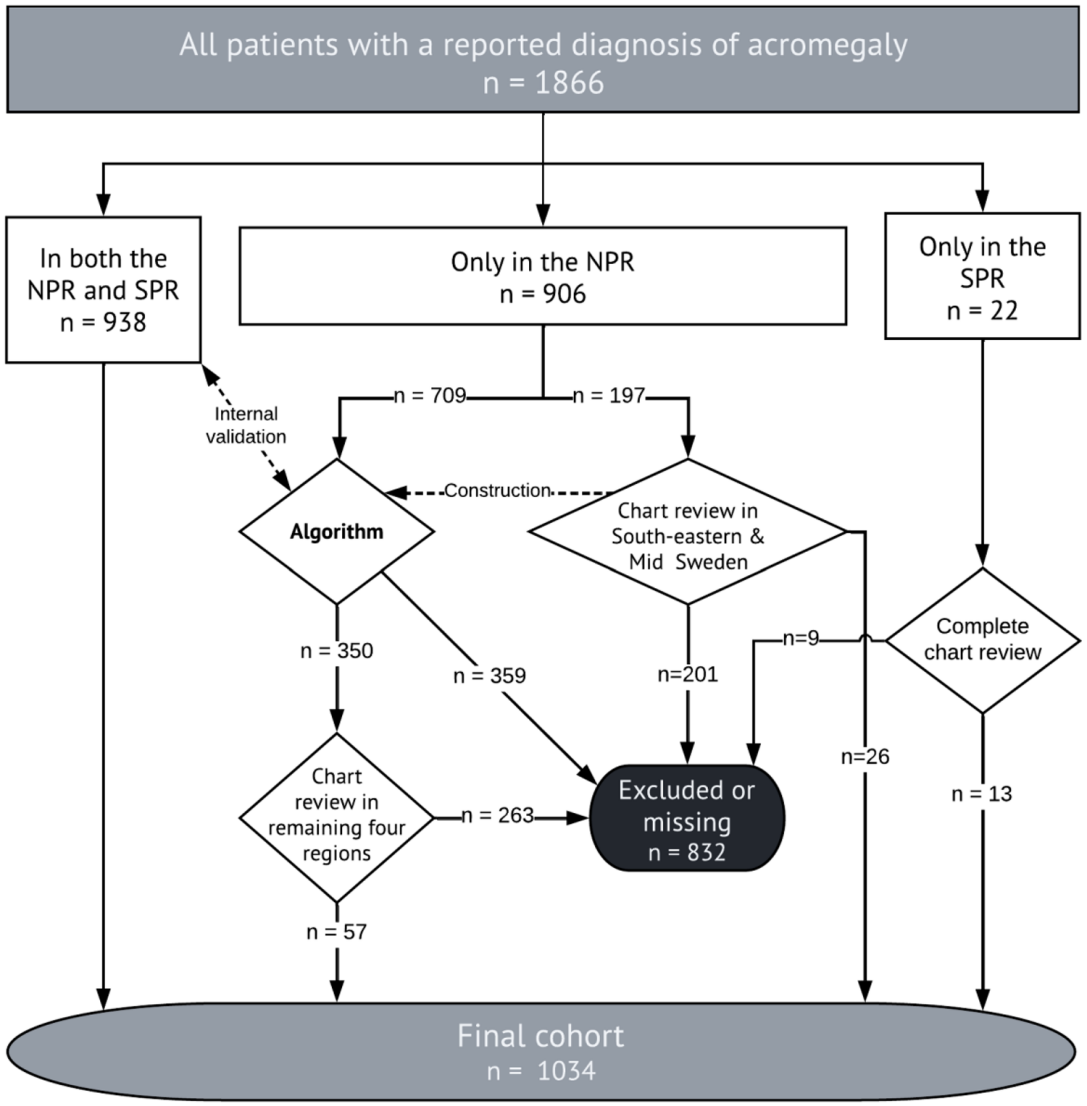
Flowchart of the process to find the final cohort of patients with a diagnosis of acromegaly from 1991 to 2018. All cases in both registers were included. Chart review was performed for patients in the SPR only group. For patients in the NPR only group, a review of medical records was performed in the south-eastern and mid Sweden health care regions. Data was used to construct an algorithm to reduce the number of false-positive cases. For the patients selected by the algorithm, a review of medical records was performed in the remaining four regions

The search in both registers generated 2355 cases with a diagnosis registration of acromegaly. A total of 489 were excluded due to age < 18 years (N = 50), first diagnosis before 1991 (N = 426), or both (N = 11), or due to incomplete data (N = 2). Among the remaining 1866 cases, 938 were found in the NPR + SPR group and were therefore considered true cases as per the definition; 22 cases were found in the SPR only group, and 906 cases were found in the NPR only group (Fig. 2).

### Construction and validation of the algorithm

The construction of the algorithm was based on patient data from the south-eastern and mid Sweden health care regions, representing one third of the Swedish population. In the NPR only group, seven out of 76 patients in south-eastern Sweden and 19 out of 121 patients in mid Sweden were confirmed to have acromegaly after a review of medical records (Fig. 2). Data on individual variables were used to construct the algorithm (Table 1). Patient visits to university hospitals with an acromegaly diagnosis as a variable was omitted from the algorithm since it increased the population to validate to 46% while only marginally increasing sensitivity. To avoid missing recently diagnosed patients, we allowed the inclusion of patients with only one registration during or after 2017, as these patients may not have had the time for a second registered visit. The algorithm is presented below.

- Patients with a first diagnosis before 2017 were included if they fulfilled one of the following criteria:
  - At least two registrations with the diagnosis code 253A (ICD-9, used before 1997)
  - At least one registration with the diagnosis code 253A and at least one of the ICD-9 diagnosis codes for pituitary tumours (225A, 225X, 227D or 237A)
  - At least two registrations, with at least one with code E22.0 (acromegaly in the ICD-10, used after 1997) as a primary diagnosis
  - At least one registration with the diagnosis code E22.0 and at least one of the ICD-10 diagnosis codes for pituitary tumours (D33.0, D33.7, D33.9, D35.2 or D44.3)
- Patients with a first diagnosis in 2017 or 2018 were included if they fulfilled one of the following criteria:
  - At least one registration with the diagnosis code E22.0 as a primary diagnosis
  - At least one registration with the diagnosis code E22.0 and one of the ICD-10 diagnosis codes for pituitary tumours (D33.0, D33.7, D33.9, D35.2 or D44.3)
- Patients with a first diagnosis any year between 1991 and 2018 were included if they fulfilled the following criteria:
  - At least one registration with the diagnosis code E22.0 or 253A and the AAE10 code for pituitary surgery used in the Classification of Care Measures (KVÅ) register.

**Table 1:** Patients from the NPR only group in south-eastern and mid Sweden health care regions, i.e., the cases that were used to construct the algorithm for selection of patients to review in the rest of Sweden

	Confirmed acromegaly (n)		PPV (%) (95% CI)	Cases lost (n)	Sensitivity (%) (95% CI)
	Yes	No			
All valid cases	26	140	15.7 (10.5–22.1)	0	100
≥ 2 diagnosis registrations	19	60	24.1 (15.1–35)	7	73.1 (52.2–88.4)
≥ 3 diagnosis registrations	16	45	26.2 (15.8–39.1)	10	61.5 (40.6–79.8)
≥ 4 diagnosis registrations	15	40	27.3 (16.1–41)	11	57.7 (36.9–76.6)
Pituitary surgery	5	0	100	22	19.2 (6.6–39.4)
Visit to a university hospital	14	66	17.5 (9.9–27.6)	12	53.8 (33.4–73.4)
Primary diagnosis of acromegaly	19	105	15.3 (9.5–22.9)	7	73.1 (52.2–88.4)
Tumour diagnosis: D35.2 or 227D	6	17	26.1 (10.2–48.4)	20	23.1 (9–43.6)
Tumour diagnosis: D44.3 or 237A	6	0	100	20	23.1 (9–43.6)
Algorithm	23	66	25.8 (17.1–36.2)	3	88.5 (69.8–97.6)

The table shows the effect of single conditional filters on PPV for confirmed acromegaly, the number of true cases that are lost when using the filter, and sensitivity. The algorithm is a combination of these filters

*PPV* positive predictive value, *NPR* National Patient Register

The algorithm was validated by applying it to the cases in the NPR + SPR group, in which the cases were considered true as per the case definition. The sensitivity of the algorithm was 98.9% (95% CI 98.1–99.5) in identifying patients in the NPR + SPR group (928 of 938 patients).

### Patients with an acromegaly diagnosis in the NPR

After applying the algorithm to the patients in the NPR only group in the four regions outside the two health care regions where all acromegaly diagnoses were validated by review of medical records, 350 of 709 patients (49.3%) were selected for review of medical records. After review of medical records in these four regions, the diagnosis of acromegaly was confirmed in 57 and rejected in 142 of these cases (Fig. 2).

Across all regions, there were a total of 146 cases where the diagnosis could not be confirmed or rejected, and five cases where the initial diagnosis was made outside of Sweden. Consequently, these 151 cases were excluded from further analysis. Among the 1844 patients originally in the NPR (groups NPR + SPR and NPR only), 1693 cases remained after exclusion of the 151 cases above and 1021 (60.3%) were confirmed as cases of acromegaly diagnosed during the study period (1991–2018). An additional 100 cases that had a true acromegaly diagnosis before 1991, but their first records in the NPR after 1991 were found during a review of medical records, bringing the number of acromegaly cases in the NPR during the study period to 1121 (66.3% of the initial 1693).

An evaluation of several limiting filters was performed for all patients in the case cohort that were registered in the NPR (i.e., NPR only and NPR + SPR groups). The most sensitive filters were ≥ 2 diagnosis registrations (sensitivity 96.3% [95% CI 94.9–97.4%]), primary diagnosis of acromegaly (93.2% [91.5–94.7%]) and visit to a university hospital (90.9% [89.0–92.6%]). The most highly predictive filters were pituitary surgery (810 true cases of 814 filter hits, 99.5%) and ICD-10 diagnosis of benign pituitary tumour (734 of 796, 92.2%).

A review of medical records identified 212 cases in the NPR only group that had a diagnosis other than acromegaly (Table 2) and were thus removed from the final cohort.

**Table 2:** Reasons for incorrect diagnosis or any alternate diagnosis among patients with an ICD code for acromegaly in the NPR only group

Diagnosis	N	%
Suspected but disproven acromegaly	40	18.9
Hyperprolactinemia/prolactinoma	51	24.1
Nonfunctioning pituitary adenoma	19	9.0
Other/unknown pituitary tumours or cysts	26	12.3
Unrelated diagnosis	48	22.6
Excess GH with nonpituitary origin	4	1.9
Unknown reason/diagnosis	24	11.3
Total	212	100%

### Coverage of the SPR

Among all 1034 patients in the final cohort (in the NPR, SPR or both), 951 (92.0%) were registered in the SPR; see Table 3 for SPR coverage in different regions. Thirteen of these 1034 patients (1.3%) were registered in the SPR but not in the NPR. Nine of the 22 patients in the SPR only group had an incorrect diagnosis. Of patients who had undergone pituitary surgery according to the registers (see below), 94.2% (767 of 814) had a registered surgery in the SPR.

**Table 3:** Incidence and number of confirmed cases with sex and age distribution compared to the population per health care region

Region	Cases				Population		Incidence (95% CI) [/10^6^/years]			DoC	Missing/no chart review
N = 6	N	%	Sex (% male)	Age (years) mean (± SD)	N (× 10^6^)	%	Adults ≥ 18 years	Total population	Age-standardised	(%)	(n)
Northern	81	7.8	42	50.8 (15.3)	0.9	8.8	4.1 (3.2–5.1)	3.2 (2.6–4.0)	3.3 (2.8–3.7)	84.0	2
Stockholm/Gotland	251	24.2	55	48.4 (14.2)	2.4	23.5	5.7 (5.0–6.5)	4.5 (3.9–5.1)	4.6 (4.0–5.2)	92.4	12
South-eastern	111	10.8	49	53.0 (14.7)	1.1	10.4	5.1 (4.2–6.1)	4.0 (3.3–4.8)	4.1 (3.6–4.7)	93.7	10
Southern	224	21.8	46	54.1 (15.2)	1.9	18.2	6.2 (5.4–7.0)	4.9 (4.2–5.5)	5.0 (4.4–5.6)	92.9	9
Mid Sweden	218	21.0	52	53.6 (16.0)	2.1	20.6	5.0 (4.4–5.7)	4.0 (3.5–4.5)	4.0 (3.5–4.5)	91.3	20
Western	149	14.4	53	49.5 (14.4)	1.9	18.6	4.0 (3.4–4.7)	3.1 (2.6–3.7)	3.1 (2.7–3.6)	94.0	93
Total	1034	100	51	51.5 (15.1)	10.2	100	5.1 (4.8–5.4)	4.0 (3.8–4.3)	4.1 (3.9–4.3)	92.0	146

The 'Missing/No Chart Review' column quantifies the cases in each region that either couldn't be validated or were not reviewed during the examination of medical records. Populations of health care regions at the end of 2018. Population data from Statistics Sweden. Incidence calculated with the adult population and with the total population contributing to years at risk. Age-standardised incidence was calculated with the total population as the denominator and was standardised to Sweden’s population at the year 2000

*DoC* degree of coverage for the SPR

In the NPR + SPR group, 92.8% (870 of 938 patients) had at least one follow-up form registered in the SPR.

### Final cohort and incidence of acromegaly in Sweden from 1991 to 2018

**Fig. 3:**
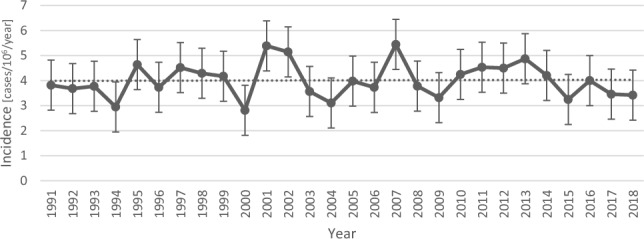
Graph of the yearly crude incidence rate of acromegaly in Sweden from 1991 to 2018 with 95% CIs, using the total background population. Fitted linear equation: yearly incidence = 0.001 year + 4.010, P (yearly change) = 0.88

The final cohort consisted of 1034 patients with acromegaly; 50.6% were men (524 of 1034), and the mean age at diagnosis was 51.5 years (18–91 years, SD ± 15.1 years). The mean age was 53.5 years (SD ± 15.9 years) in women and 49.7 years (SD ± 14.1 years) in men. Regional distribution, as determined by hospital location at first diagnosis, is shown in Table 3. In 67.8% (N = 701) of the patients, the first acromegaly diagnosis was received at a university hospital, while 90.1% (N = 940) had received at least one diagnosis at a university hospital. The national crude incidence rate of acromegaly in the population for the period 1991–2018 was 4.0 cases per million per year (95% CI 3.8–4.3), including individuals < 18 years of age in the at-risk population. No significant increase or decrease in incidence was observed during the study period (P = 0.88; Fig. 3). Including only adults (≥ 18 years) as the population at risk yielded an incidence rate of 5.1 cases per million per year (95% CI; 4.8–5.4) (Table 3). For comparison purposes, using the Swedish 2000 standard population or the WHO standard population as a reference resulted in an incidence rate of 4.1 (3.9–4.3) and 4.0 (3.9–4.1), respectively.

Pituitary surgery was performed at least once in 78.7% (N = 814) of patients, defined as having the AAE10 CCM code or having a registered surgery form in the SPR. There was no significant difference in the frequency of surgery before and after 2007 when reporting in the CCM became obligatory (80.6% vs. 76.4%, P = 0.1).

## Discussion

By combining the Swedish Pituitary Register with the National Patient Register, we established a valid national cohort of patients diagnosed with acromegaly for the period 1991–2018. An algorithm was developed and tested with 99% sensitivity among patients with acromegaly reported in both registers and halved the number of patients who needed diagnosis confirmation through review of medical records in the NPR only group. After evaluation, two-thirds of the acromegaly diagnoses in the NPR were confirmed, either through the case definition or review of medical records. Ninety-two percent of the patients in the final cohort were reported in the more disease-specific but less sensitive SPR.

The incidence rate of 4.0 cases per million per year was calculated using the entire population as individuals at risk. Thus, since we did not include individuals < 18 years of age in the present study, the calculated incidence rate will likely be falsely low, as the disease is less common among children [11]. Conversely, an incidence calculated with only adults as both at-risk and case populations will yield a rate of 5.1 cases per million per year. It is likely that the latter incidence rate may be higher compared to other studies that have also included subjects < 18 years of age in the case population.

For comparison purposes we also computed standardised incidence rate with both Sweden 2000 and WHO 2000 as standard populations. In the present study, the crude incidence rate of 4.0, as well as the age standardised rates of Sweden 2000 (4.1) and WHO 2000 (4.0) were all higher compared to those of previous studies in Sweden (3.5–3.7) [9, 10], northern Finland (3.4) [12] and Denmark (3.8) [13]. Among Scandinavian countries, only Iceland has reported an increasing incidence rate (7.7), but only after year 2005 [14]. A review and meta-analysis of 32 studies reported an incidence rate of 3.8 [15]. In addition, a recent Danish systematic literature review of 31 studies revealed an incidence rate ranging from 2 to 4 cases/10^6^ to 4 to 5 cases/10^6^ during more recent study periods [16]. The highest incidence was mainly found in small populations with well-defined catch-up areas and centralized treatment which was in line with the reported incidence of 4.6 cases/10^6^ found in patients in the North Denmark Region during 1992–2021 [16].

The incidence rate varied between the different regions in Sweden, with the Stockholm/Gotland and southern health care regions having the highest rates and the northern and western health care regions having the lowest rates. The northern region is sparsely populated in comparison with the more densely populated Stockholm/Gotland and southern regions, with long distances to health care facilities and thus perhaps a higher threshold for seeking medical care. The incidence rate in the western region was in line with a previous thorough incidence study from that region [10].

Our data suggest that, concerning the acromegaly diagnosis, a review of medical records or a more stringent combination of criteria when extracting cases from the NPR is required before using any data from patient registers based on ICD codes. After a review of the medical records of all patients with a diagnosis of acromegaly in the Danish Patient Register, a PPV of 66.7% [17] was found, which is in line with the findings in the present study.

To increase the number of patients with a diagnosis of acromegaly, we used different limiting filters on patient data in the NPR. The most specific filters were pituitary surgery and, to a lesser extent, pituitary tumour diagnoses. The most sensitive filters were more than one registration, primary diagnosis of acromegaly and visit to a university hospital. A Danish study showed similar results, with more than one diagnosis registration and visit to a university hospital increasing PPV while maintaining a high sensitivity [17]. A combination of these filters can be used for final patient selection or selection of cases to validate by review of medical records, depending on the type of study.

Using variables from the NPR, such as pituitary tumour and surgery, for patient selection is not a novel approach. Several studies, including some on acromegaly, have developed algorithms with high specificity to identify true cases nationwide or in large regions [18–21]. It is therefore conceivable that the size of the case cohorts may have been underestimated in previous studies, which may have affected the outcomes. In the present study, we used an algorithm with high sensitivity in the first stage to reduce the number of patients for review of medical records in the second stage. This approach allowed us to provide data with good completeness while still markedly reducing the need for review of medical records, which is of great importance for future studies on acromegaly and comorbidity.

Internal validation of the algorithm yielded almost 99% sensitivity. Both the Stockholm/Gotland and southern health care regions had higher incidence rates than the mid Sweden and south-eastern health care regions; in the latter, every single acromegaly diagnosis registration in the NPR only had been manually reviewed. This provides further proof of the sensitivity of the algorithm.

We found that 92% of the patients in the final cohort of patients with acromegaly were registered in the SPR. The median age, distribution of men/women and proportion of patients who had undergone surgery in the whole study population were in accordance with the results of a recently published study among patients with acromegaly reported in the SPR between 1991 and 2011 [9].

The absence of validation of the patients registered in both the NPR and SPR, considered true as per the case definition, is a limitation and posed a risk for including false-positives. However, our main hypothesis was that the diagnosis of acromegaly was considered correct for patients identified in both registers and the risk for false positive diagnoses were estimated to be low. The reasons for this are that we assume that SPR have a high specificity of the acromegaly diagnosis. Data is manually reported in the SPR, such as hormone values, treatment (surgery, radiotherapy, pharmacological treatment specific for acromegaly) as well as biochemical control. Hormone values for GH and IGF-1 can only be reported for patients with an acromegaly diagnosis in the SPR. Monitors have previously manually checked data and the register has an automatic logic check system to reduce incorrect data. Furthermore, 93% of the patients had at least one follow-up form registered in the SPR, with diagnosis specific data, further confirming the diagnosis.

The Classification of Care Measures register records started in 2007. Records of any surgeries performed before 2007 that are not present in the SPR may not be available. This may lead to an underestimation of the number of performed surgeries. However, the surgery rates are similar among patients diagnosed before and after 2007.

There were 146 patients for which no medical records could be found or for which a review of medical records was not performed. These patients were excluded from the analysis, although at least some of them may have had a correct diagnosis of acromegaly which may have affected the results.

The use of the algorithm itself is of course inferior in comparison to manually reviewing all patients who have received the acromegaly diagnosis in the context of data completeness and validity. The algorithm has shown reliable results in validation, and it is unlikely that the small number of true cases that may have been lost would have affected our results in any major way.

We searched the NPR for acromegaly diagnoses set after 1987 to exclude patients diagnosed between 1987 and 1991, thus avoiding these cases being rediscovered during the review process. However, several patients diagnosed before 1991 were still discovered during the evaluation, indicating that they either were not admitted to the hospital at the time of the first diagnosis or were admitted before 1987 but not between 1987 and 1991.

## In conclusion

By combining the Swedish Pituitary Register and the National Patient Register, the cohort of patients with a correct diagnosis of acromegaly was improved. The final valid cohort provided an improved estimate of the incidence of acromegaly in Sweden. The diagnostic code for acromegaly alone is not sufficient to identify patients in the NPR. Furthermore, the use of an algorithm reduced the number of patients that needed validation through review of medical records, and is a reliable way to improve the accuracy of the diagnosis in registers based on ICD codes in future studies.

## Data Availability

The data generated or analyzed during this study are available from the corresponding author upon reasonable request.
